# Lactate Predicts Neurological Outcomes after Perinatal Asphyxia in Post-Hypothermia Era: A Prospective Cohort Study

**DOI:** 10.3390/life11111193

**Published:** 2021-11-05

**Authors:** Yi-Fang Tu, Po-Ming Wu, Wen-Hao Yu, Chung-I Li, Cheng-Lin Wu, Lin Kang, Yung-Chieh Lin, Hsin-I Shih, Chao-Ching Huang

**Affiliations:** 1 Department of Pediatrics, National Cheng Kung University Hospital, College of Medicine, National Cheng Kung University, Tainan City 70403, Taiwan; bradsteve41@gmail.com (P.-M.W.); fieldof19@yahoo.com.tw (W.-H.Y.); appledr@gmail.com (Y.-C.L.); 2 Institute of Clinical Medicine, College of Medicine, National Cheng Kung University, Tainan City 70101, Taiwan; wujl.towalkwithwings@gmail.com; 3 Department of Statistics, College of Management, National Cheng Kung University, Tainan City 70101, Taiwan; cili@mail.ncku.edu.tw; 4 Department of Pathology, National Cheng Kung University Hospital, College of Medicine, National Cheng Kung University, Tainan City 70403, Taiwan; 5 Department of Obstetrics and Gynecology, National Cheng Kung University Hospital, College of Medicine, National Cheng Kung University, Tainan City 70403, Taiwan; kanglin@mail.ncku.edu.tw; 6 Department of Emergency Medicine, National Cheng Kung University Hospital, College of Medicine, National Cheng Kung University, Tainan City 70403, Taiwan; n506594@mail.hosp.ncku.edu.tw

**Keywords:** perinatal asphyxia, lactate, neurological outcomes, neonatal hypoxic-ischemic encephalopathy

## Abstract

Background: Neonatal hypoxic-ischemic encephalopathy (HIE) is the most common cause of mortality and neurological disability in infancy after perinatal asphyxia. Reliable biomarkers to predict neurological outcomes of neonates after perinatal asphyxia are still not accessible in clinical practice. Methods: A prospective cohort study enrolled neonates with perinatal asphyxia. Biochemical blood tests and cerebral Doppler ultrasound were measured within 6 h of age and at the 4th day old. Neurological outcomes were assessed at 1 year old. Results: Sixty-four neonates with perinatal asphyxia were enrolled. Fifty-eight (90%) had hypoxic-ischemic encephalopathy (HIE) including 20 (34%) Stage I, 21 (36%) Stage II, and 17 (29%) Stage III. In the asphyxiated infants without therapeutic hypothermia, HIE stage, PH, and base excess levels within 6 h of age were the predictors of adverse outcomes. In the asphyxiated infants receiving therapeutic hypothermia, HIE stage failed to predict outcomes. Instead, blood lactate levels and pulsatility index (PI) of medial cerebral arteries (MCA) either in 6 h of age or at the 4th day old independently predicted adverse outcomes. Conclusions: Blood lactate, which is a common accessible test at the hospital and MCA PI on cerebral ultrasound could predict adverse outcomes in asphyxiated infants receiving therapeutic hypothermia.

## 1. Background

Perinatal asphyxia occurs in 1–1.5% of live births in developed countries, and higher in developing countries [1,2]. It is an important cause of acquired neonatal brain injury in term neonates leading to neonatal hypoxic-ischemic encephalopathy (HIE), which is the most common cause of death and neurological disability in human neonates [3,4,5]. In infants with HIE, the overall mortality was 15–25%, and up to 1/3 survivors tend to develop long-term neurological disabilities such as mental retardation, cerebral palsy, and epilepsy [5,6]. Hypothermic treatment involves inducing the neonatal body to 33–34 °C for 72 h. In the last two decades, therapeutic hypothermia has increased the rate of survival, and decreased the prominence of disability after treatment for those ages 18–24 months [6,7,8,9,10,11,12]. However, there is still a 40–50% disability in moderate/severe HIE infants after receiving therapeutic hypothermia [3,13]. Early prognostication remains challenging but essential for parental counseling and intensive care management, including the use of further neuroprotective strategies [14].

A wide variety of biomarkers from the body fluid, and neurophysiologic or neuroimage modalities had been tried to predict neurological outcomes in HIE patients. The biomarkers from body fluid included neuron specific enolase (NSE), ubiquitin carboxy-terminal hydrolase L1 (UCHL-1), brain derived neurotrophic factor (BDNF), S100B protein, glical fibrillary acidic protein (GFAP), Tau protein, inflammatory cytokines/chemokines, and so on [15,16]. The neurophysiologic or neuroimage modalities included electroencephalography (EEG), amplitude-integrated EEG (aEEG), evoked potentials, different magnetic resonance imaging (MRI) modalities, and cranial ultrasound [14]. Currently, no reliable body-fluid-based biomarkers are available in clinical practice to predict outcomes in newborns after perinatal asphyxia. The assessments of evolving brain injury and estimates of neurologic outcomes majorly rely on clinical examination, aEEG/EEG background severity, and MRI.

In addition to clinical examination, aEEG/EEG and MRI, several hospital-based conventional biochemistry blood tests such as serum PH, bicarbonate, lactate, creatine kinase (CK), Troponin-T, alanine transaminase (ALT), and lactate dehydrogenase (LDH) are hypoxia-associated markers and are accessible to most clinicians. For example, time-weighted mean serum lactate values have been used in predicting short-term or long-term outcomes after out-hospital cardiac arrest, which also caused hypoxic-ischemic impacts on the brain [17]. Moreover, bedside available ultrasound is also used widely in neonatal practice and helpful for assessing the evolution of injury and providing predictive information on the long-term neurodevelopmental outcome of newborns with brain injury [18]. In this study, we tried to use these hospital-based accessible tests to predict neurologic outcomes at 1 year in newborns after perinatal asphyxia.

## 2. Methods

This is a prospective cohort study that included all the newborns admitted with perinatal asphyxia and had a gestational age of at least 35 weeks from 2015 to 2019 in the neonatal intensive care unit of a tertiary referral medical center. Perinatal asphyxia was diagnosed if at least two of the following criteria were met: (a) Apgar score less than 5 at 5 min of age; (b) a blood base excess (deficit) of greater than −10 mmol/L during the first hour of life; (c) endotracheal intubation and intermittent positive pressure ventilation for ongoing resuscitation at 10 min of age; (d) multiorgan failure within 24 h of age; (e) evidence of fetal distress as indicated by thick meconium stained liquor and/or abnormal cardiotocographic changes (sustained fetal bradycardia < 100 beats/min, late deceleration with loss of variability and/or severe recurrent deceleration with loss of variability) [19]. Infants received therapeutic hypothermia when they fulfilled the entry criteria as the NICHD trials [3]. Cases with congenital heart diseases, major central nervous system malformations, severe growth restriction (birth weight of <1800 gm), and neonatal sepsis were excluded. Data of demographic background and medical information during the prenatal, perinatal, and postnatal periods as well as maternal information were collected after obtaining the parents’ consent.

### 2.1. Biomarker Assessments

After admission, all infants underwent a series of standardized neurologic examinations performed by pediatric neurologists. Hospital-based available biochemistry blood tests including blood gas (PH, bicarbonate, base excess), lactate, ALT, aspartate aminotransferase (AST), CK, Troponin-T, ammonia, and LDH were performed at least within 6 h of age (acute post-injury period before hypothermia therapy) and at the 4th day old (86–92 h, after rewarming if receiving therapeutic hypothermia).

Cerebral ultrasonography was also carried out within 6 h of age and at the 4th day old to rule out major congenital or traumatic abnormalities and to identify flow velocity (peak systolic and endo-diastolic), resistance index (RI), and pulsatility index (PI) using a digital ultrasound device Xario SSA-680A (Toshiba) with a sector 6–10 MHz transducer and a linear 7–14 MHz transducer. Blood flow parameters were measured in the branch of anterior cerebral arteries (ACA) located in front of the genus of the corpus callosum and the proximal branch of the right medial cerebral arteries (MCA).

### 2.2. Outcome Measurements

Early neurodevelopmental outcomes were assessed at age 1 year. The pediatric neurologists evaluated the children’s neuromotor conditions and the pediatric psychologists evaluated neurodevelopment using Bayley-Scales of Infant and Toddler Development, 3rd edition (BSID-III), which providing distinct scores for cognitive, language, and motor functions [20]. Adverse outcomes were defined as death or severe neurological disability when BSID-III developmental scores were <70 on any of the three cognitive, language, or motor domains or presence of deafness, blindness, or cerebral palsy.

### 2.3. Statistics

Data were represented as mean (standard deviation). Risk factors in biochemistry variables and parameters from Doppler ultrasound for adverse outcomes were examined by using univariate binary logistic regression analysis. Odds ratio (OR), as well as 95% confidence intervals (CIs) were calculated. The potential risk factors with significance levels of *p* < 0.05 were entered into a multivariable logistic regression model to evaluate the independent associations with adverse outcomes. A receiver operating characteristic (ROC) analysis was constructed to determine the best cut-off value to predict the outcome. The probability was calculated using a logistic regression model, and the estimated probabilities were used in a ROC analysis to calculate the area under curve (AUC) for different models. A *p* value of < 0.05 was considered statistically significant for all tests. All analyses were performed by using SPSS version 17 (IBM SPSS Statistics, IBM Corporation, Armonk, NY, USA).

## 3. Results

Sixty-four neonates with perinatal asphyxia were enrolled. The mean gestational age of them was 37.9 ± 1.4 weeks, the mean birth body weight was 2847 ± 485 g, and the gender ratio (male:female) was around 1:1 (Table 1). The mean Apgar score was 3 at 1 min and 5 at 5 min of age. Of the 64 asphyxiated infants, 58 (90%) had HIE including 20 (34%) Stage I, 21 (36%) Stage II, and 17 (29%) Stage III. Adverse outcomes occurred totally in 16 (25%) asphyxiated infants including 10 (16%) with mortality and 6 (9%) with severe disability (Table 1). Among these asphyxiated infants, 32 neonates did not receive therapeutic hypothermia. The majority of these neonates without therapeutic hypothermia were neonates with Stage I HIE (19) or without HIE (6), and the minority was those whose parents refused to receive hypothermia or who had unstable vital signs (Table 1).

The risk for adverse outcomes was analyzed among the variables from biochemistry blood tests and Doppler ultrasound within 6 h of age and at the 4th day old (Table 2 and Table 3). In the asphyxiated infants without therapeutic hypothermia, PH (*p* = 0.015) and base excess (BE) (*p* = 0.023) levels within 6 h of age and BE levels (*p* = 0.026) at the 4th day old as well as the HIE stage (*p* = 0.006) were the risk variables for adverse outcomes. On the other hand, several risk variables were significant in the asphyxia neonates who received therapeutic hypothermia. Among biochemistry blood tests, PH (*p* = 0.008), BE (*p* = 0.009), lactate (*p* = 0.015), and ammonia (*p* = 0.036) levels within 6 h of age (Table 2) and lactate (*p* = 0.011) at the 4th day old were at risk for adverse outcomes (Table 3). Among Doppler ultrasound, the diastolic velocity (*p* = 0.026), RI (*p* = 0.012), and PI of MCA (*p* = 0.015) within 6 h of age (Table 2), and all variables except RI of MCA and PI of ACA at the 4th day old were at risk for adverse outcomes (Table 3). When taking these risk variables into the multivariable analysis, we selected two risk variables (one from biochemistry blood tests, and one from Doppler ultrasound parameters) in order to avoid overfitting and interactions. We also prefer to choose the repetitive significant risk variables at both time points. Finally, we found that lactate and PI of MCA either within 6 h of age (lactate: OR: 1.28, 95% CI: 1.00–1.63, *p* = 0.046; PI: OR: 0.04, 95% CI: 0.003–0.59, *p* = 0.019) or at the 4th day old (lactate: OR: 5.31, 95% CI: 1.16–24.43, *p* = 0.032; PI: OR: 0.002, 95% CI: 0.00–0.69, *p* = 0.037) were consistently at risk for adverse outcomes among asphyxiated infants who received therapeutic hypothermia (Table 4).

**Figure 1:**
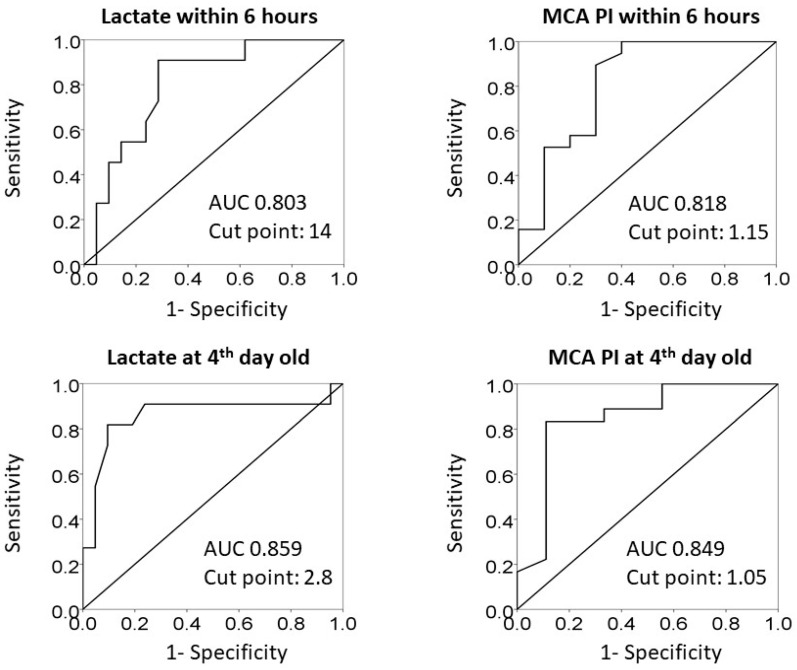
ROC curve analysis for the efficacy of lactate and pulsatility index of medial cerebral arteries for predicting adverse outcomes.

The optimum cut-off values for lactate and PI of MCA in the asphyxiated infants who received therapeutic hypothermia were identified by drawing ROC curves (Figure 1). The area under ROC curves (AUC) and cut-off values were shown in Table 5. Within 6 h of age, the cut-off value of lactate was 14 mmol/L (*p* = 0.015) and PI of MCA was 1.15 (*p* = 0.015). At the 4th day old, the cut-off value of lactate was 2.8 mmol/L and PI of MCA was 1.05. The sensitivity and specificity of either lactate (*p* = 0.011) or PI of MCA (*p* = 0.011) at the 4th day old were better than that within 6 h of age (Table 5).

## 4. Discussion

We demonstrate the ability of some bed-side available, relatively objective examinations in asphyxiated infants during the acute postnatal period to severe as the early predictors of adverse neurodevelopmental outcomes at 1 year old. For asphyxiated infants who did not receive or indicate therapeutic hypothermia, the particular predictor was the initial HIE stage within 6 h of age (OR: 16.5, 95% CI: 2.19–124.10, *p* = 0.006). In contrast, the initial HIE stage was not a predictor of adverse outcomes for asphyxiated infants who received therapeutic hypothermia. The lactate and pulsatility index of MCA either within 6 h of age (lactate: OR: 1.28, 95% CI: 1.00–1.63, *p* = 0.046; PI: OR: 0.04, 95% CI: 0.003–0.59, *p* = 0.019) or after rewarming from hypothermia at the 4th day old (lactate: OR: 5.31, 95% CI: 1.16–24.43, *p* = 0.032; PI: OR: 0.002, 95% CI: 0.00–0.69, *p* = 0.037) became the independent predictors of adverse outcomes. The cut-off point of lactate was 14 and 2.8 mmol/L and of MCA PI was 1.15 and 1.05, respectively, measured within 6 h of age and at the 4th day old.

It is known that lactate is a common product of glycolysis, an anaerobic metabolic pathway. Hence, it was prominent why lactate levels increase when oxygen levels decrease and/or when tissues underwent hypoperfusion. In the situations of hypoperfusion or hypoxia, pyruvate will no longer enter into the mitochondria for aerobic metabolism, instead, it is preferentially reduced to lactate, resulting in the accumulation of lactate in the blood [21]. As blood lactate can be easily and quickly determined, it has been used as a surrogate of low brain oxygenation or mitochondria dysfunctions in some neurological diseases, such as traumatic brain injury and multiple sclerosis as well as neonatal HIE [22,23,24,25,26,27]. Traumatic brain injury could create mitochondrial damage and impair oxidative metabolism with lactate production. Hence, serum lactate on admission was reported to strongly suggest severe injury and to predict in-hospital mortality in pediatric patients with traumatic brain injury [22,24]. In multiple sclerosis, alternations in mitochondria lead to diminish ATP supply, increase glycolysis, accumulate pyruvate, and produce lactate. Monitoring serum lactate could reflex “virtual hypoxia” and therapeutic outcomes in multiple sclerosis [23]. In the neonatal population, elevated postnatal lactate levels have been described as a risk factor indicating the possible onset and development of severe, postpartum asphyxia [25]. Postnatal hyperlactatemia has also been established to correlate with the severity of HIE as well as neurological morbidity and mortality in the first days of life [26,27,28]. However, postnatal lactate as a potential predictor of the long-term outcome of asphyxiated infants has not yet been comprehensively studied, particularly in the post-hypothermia era [29]. We observed that lactate either within 6 h of age or after rewarming from hypothermia at the 4th day old is the independent predictor of adverse outcomes in asphyxiated infants who received therapeutic hypothermia. In accordance with our findings, studies of Polackova et al. and Chiang et al. included asphyxiated infants who had moderate to severe HIE and received therapeutic hypothermia and showed lactate levels at 3, 6, 12, 24, 36 h of age, or after 72 h of therapeutic hypothermia were significantly higher in those with adverse outcomes at 2 years old compared with those with favorable outcomes [29,30]. In our study, we had a larger sample size (64 infants) compared with these two previous studies (51 and 17 infants, respectively), and we further could offer the cut-off value of lactate (14 mmol/L within 6 h of age; 2.8 mmol/L at the 4th day old after hypothermia) to aid in predicting adverse outcome in clinical practice. In addition, we included all asphyxiated infants either with or without therapeutic hypothermia. Hence, our findings illustrated that, on the other hand, lactate would not predict outcomes in asphyxiated infants without therapeutic hypothermia.

Doppler ultrasound with spectral analysis of the cerebral blood flow is a safe, bed-side available, and cost-efficient modality to measure neonatal cerebral hemodynamic status following HIE [31]. After asphyxia, the hyperemic phase with cerebral vasodilatation resulting in a fall of vascular resistance is responsible for secondary brain injury. Through measuring cerebral vascular changes from the ACA and MCA, several Doppler parameters including cerebral blood flow velocities and RI particularly at the age of 12 ± 2 h had been known to serve as an early predictor for neuromotor outcomes in the asphyxiated infants in the pre-hypothermia era [32,33,34]. Nonetheless, recent studies showed that hypothermia makes RI a poor predictor unless it was measured after rewarming from 72 h of therapeutic hypothermia when it could regain the predictive power for adverse outcome [35,36]. In contrast, we found that either within 6 h of age or at the 4th day old after rewarming, the PI of MCA instead of cerebral blood flow velocities or RI is the independent predictor of adverse outcome in asphyxiated infants who received therapeutic hypothermia. The reason for the PI better than RI in our study is not clear. The PI primarily depends on mean velocity, whereas the RI is mainly affected by systolic velocity [37]. It can be hypothesized that PI involves mean flow velocity that included both peak-systolic and end-diastolic flow velocity and could represent the cerebral vascular changes of the entire cardiac cycle better than systolic velocity only. It may be similar to the fact that mean blood pressure is a better indicator of perfusion of vital organs compared with systolic blood pressure and a better predictor of outcome in critical patients [38].

There are some limitations to the study. The blood samples in our study were mostly taken from umbilical veins through catheters to minimize the regional tissue hypoxia effects on the lactate levels during sampling. Usually, the cerebral ultrasound was performed while the vital signs of these asphyxiated babies were relatively stable to minimalize the systemic circulation influences on the cerebral blood flow. The neurological outcomes are difficult to be measured in young children only by physical examinations at clinics. Hence, we quantify the outcomes by using BSID-III, which is the most commonly used psychometric instrument by health care professionals [39]. Our findings will still require validation in a larger cohort.

## 5. Conclusions

Blood lactate, which is a common accessible test at the hospital and MCA PI on cerebral ultrasound in as early as 6 h of age could predict adverse outcomes in asphyxiated infants receiving therapeutic hypothermia.

## Figures and Tables

**Table 1:** Characteristics of study patients.

	Non-Hypothermia *n* = 32	Hypothermia *n* = 32	*p* Value	Total *n* = 64
Gestational age	38.0 ± 1.5	37.7 ± 1.4	0.430	37.9± 1.4
Gender (M/F)	17/15	14/18	0.617	31/33
Birth body weight (gm)	2789 ± 507	2905 ± 462	0.165	2847 ± 485
HIE			<0.001	
Stage 1	19	1		20
Stage 2	3	18		21
Stage 3	4	13		17
No HIE	6	0		6
Apgar Score				
1 min	3 ± 2	2 ± 2	0.050	3 ± 2
5 min	5 ± 2	4 ± 3	0.095	5 ± 2
Vital signs on admission				
Systolic blood pressure	64 ± 10	68 ± 15	0.172	
Diastolic blood pressure	39 ± 11	40 ± 10	0.514	
Heart rate	144 ± 20	141 ± 27	0.588	
Sentinel events			0.129	
Uterine rupture	0	5		5
Placenta abruption		5		8
Shoulder dystocia	1	0		1
Maternal complications			0.032	
Preeclampsia/eclampsia	0	4		4
PIH	1	6		7
GDM	2	2		4
Outcomes				
Death	3	7	0.302	10
Severe disability	2	4	0.672	6

HIE: hypoxic-ischemic encephalopathy; PIH: pregnancy-induced hypertension; GDM: gestational diabetes mellitus.

**Table 2:** Risk of variables within 6 h for adverse neurological outcomes at 1 year old.

	No Hypothermia (*n* = 32)				Hypothermia (*n* = 32)			
	Mean ± SD	OR	95% CI	*p* Value	Mean ± SD	OR	95% CI	*p* Value
HIE stage	1 ± 1	16.52	2.19–124.10	0.006	2 ± 0			0.998
Biochemistry variables								
PH	7.18 ± 0.20	<0.001	0.00–0.17	0.015	6.98 ± 0.26	0.001	0.00–0.15	0.008
HCO3	13.8 ± 4.1			0.060	12.3 ± 4.0	-	-	0.052
BE	−14.2 ± 6.1	0.65	0.45–0.94	0.023	−18.8 ± 5.98	0.70	0.54–0.92	0.009
AST	111 ± 113	-	-	0.154	226 ± 276	-	-	0.297
ALT	32 ± 43	-	-	0.110	65 ± 73	-	-	0.276
LDH	1081 ± 1170	-	-	0.477	1995 ± 1791	-	-	0.192
Creatinine	0.82 ± 0.19	-	-	0.070	0.86 ± 0.25	-	-	0.579
Lactate	8.9 ± 2.4	-	-	0.161	12.8 ± 6.3	1.22	1.04–1.44	0.015
CK	2169 ± 5842	-	-	0.073	1534 ± 1374	-	-	0.830
TnT	0.17 ± 0.14	-	-	0.757	0.45 ± 0.55	-	-	0.330
Ammonia	52 ± 68	-	-	0.322	108 ± 129	1.02	1.00–1.04	0.036
Doppler ultrasound								
Peak velocity								
ACA	21.5 ± 8.9	-	-	0.557	20.7 ± 8.5	-	-	0.376
MCA	36.9 ± 13.4	-	-	0.438	35.0 ± 15.4	-	-	0.417
Diastolic velocity								
ACA	5.3 ± 4.1	-	-	0.492	8.1 ± 5.9	-	-	0.171
MCA	9.3 ± 6.6	-	-	0.210	14.1 ± 10.3	1.13	1.02–1.25	0.026
Resistance index								
ACA	0.76 ± 0.14	-	-	0.179	0.64 ± 0.15	-	-	0.077
MCA	0.74 ± 0.14	-	-	0.984	0.61 ± 0.17	<0.001	0.00–0.13	0.012
Pulsatility index								
ACA	1.45 ± 0.51	-	-	0.196	1.09 ± 0.55			0.201
MCA	1.50 ± 0.65	-	-	0.107	1.05 ± 0.40	0.05	0.004–0.54	0.015

ACA: anterior cerebral artery; ALT: alanine aminotransferase; AST: aspartate aminotransferase; BE: base excess; CK: creatine kinase; HCO3: bicarbonate; LDH: lactate dehydrogenase; MCA: middle cerebral artery; TnT: troponin T.

**Table 3:** Risk of variables at the 4th day old for adverse neurological outcomes at 1 year old.

	No Hypothermia *n* = 32				Hypothermia *n* = 32			
	Mean ± SD	OR	95% CI	*p* Value	Mean ± SD	OR	95% CI	*p* Value
Biochemistry variables								
PH	7.34 ± 0.23	-	-	0.053	7.38 ± 0.08	-	-	0.271
HCO3	23.1 ± 5.6	-	-	0.229	27.6 ± 4.4	-	-	0.234
BE	−2.5 ± 8.5	0.88	0.79–0.99	0.026	1.8 ± 3.9	-	-	0.171
AST	75 ± 37	-	-	0.183	150 ± 147	-	-	0.247
ALT	30 ± 34	-	-	0.070	78 ± 82	-	-	0.669
LDH	751 ± 445	-	-	0.276	1779 ± 1547	-	-	0.255
Creatinine	0.57 ± 0.28	-	-	0.731	0.71 ± 0.42	-	-	0.493
Lactate	2.3 ± 0.9	-	-	0.604	2.7 ± 2.2	2.97	1.28–6.90	0.011
CK	1016 ± 1421	-	-	0.844	2387 ± 2483	-	-	0.692
TnT	0.11 ± 0.10	-	-	0.055	0.24 ± 0.29	-	-	0.054
Ammonia	27 ± 12	-	-	0.982	29 ± 16	-	-	0.084
Doppler ultrasound								
Peak velocity								
ACA	32.4 (7.4)	-	-	0.518	30.4 (10.4)	1.13	1.02–1.26	0.023
MCA	52.1 (14.8)	-	-	0.129	55.7 (23.2)	1.11	1.02–1.20	0.012
Diastolic velocity								
ACA	10.0 (4.1)	-	-	0.894	11.3 (7.5)	1.21	1.04–1.41	0.014
MCA	16.4 (7.5)	-	-	0.507	22.7 (13.8)	1.10	1.02–1.18	0.015
Resistance index								
ACA	0.69 (0.09)	-	-	0.539	0.65 (0.16)	0.001	0.00–0.81	0.043
MCA	0.71 (0.12)	-	-	0.648	0.62 (0.14)	-	-	0.275
Pulsatility index								
ACA	1.25 (0.30)	-	-	0.362	1.11 (0.42)	-	-	0.064
MCA	1.24 (0.31)			0.643	1.07 (0.32)	0.002	0.00–0.25	0.011

ACA: anterior cerebral artery; ALT: alanine aminotransferase; AST: aspartate aminotransferase; BE: base excess; CK: creatine kinase; HCO3: bicarbonate; LDH: lactate dehydrogenase; MCA: middle cerebral artery; TnT: troponin T.

**Table 4:** Multivariable analysis among asphyxia neonates with therapeutic hypothermia.

	Univariate		Multivariable Model 1			Multivariable Model 2		
	OR	*p* Value	OR	95% CI	*p* Value	OR	95% CI	*p* Value
Significant variables with 6 h of age								
PH	0.001	0.008	-	-	-	-	-	-
BE	0.701	0.009	-	-	-	-	-	-
Lactate	1.221	0.015	1.32	1.04–1.69	0.024	1.28	1.00–1.63	0.046
Ammonia	1.019	0.036	-	-	-	-	-	-
Diastolic velocity								
MCA	1.126	0.026	1.19	1.01–1.42	0.043	-	-	-
Resistance index								
MCA	<0.001	0.012	-	-	-	-	-	-
Pulsatility index								
MCA	0.045	0.015	-	-	-	0.04	0.003–0.59	0.019
Significant variables at the 4th day old								
Lactate	2.972	0.011	4.11	1.14–14.81	0.031	5.31	1.16–24.43	0.032
Peak velocity								
ACA	1.130	0.023	-	-	-	-	-	-
MCA	1.084	0.011	-	-	-	-	-	-
Diastolic velocity								
ACA	1.211	0.014	-	-	-	-	-	-
MCA	1.066	0.046	1.05	0.965–1.15	0.254	-	-	-
Resistive index								
ACA	0.001	0.042	-	-	-	-	-	-
Pulsatility index								
MCA	0.002	0.011	-	-	-	0.002	0.00–0.69	0.037

BE: base excess; ACA: anterior cerebral artery; MCA: middle cerebral artery.

**Table 5:** Cut-off value of predictors of adverse outcome.

	AUC	Cut-Off Value	Specificity (%)	Sensitivity (%)	PPV (%)	NPV (%)	*p* Value
Significant variables within 6 h of age							
Lactate	0.803	14.0	0.625	0.938	0.714	0.909	0.015
PI of MCA	0.818	1.15	0.778	0.850	0.895	0.700	0.015
Significant variables at the 4th day old							
Lactate	0.859	2.80	0.818	0.905	0.905	0.818	0.011
PI of MCA	0.849	1.05	0.727	0.938	0.833	0.889	0.011

MCA: middle cerebral artery; NPV: negative predictive value; PI: pulsatility index; PPV: positive predictive value.

## Data Availability

The data presented in this study are available on request from the corresponding author.

## Abbreviations

ACA	anterior cerebral arteries
aEEG	amplitude-integrated electroencephalography
ALT	alanine transaminase
AST	aspartate transaminase
EEG	electroencephalography
CK	creatine kinase
HIE	hypoxic-ischemic encephalopathy
LDH	lactate dehydrogenase
MCA	medial cerebral arteries
PI	pulsatility index
RI	resistance index
